# Erectile Function, Incontinence, and Other Quality of Life Outcomes Following Proton Therapy for Prostate Cancer in Men 60 Years Old and Younger

**DOI:** 10.1002/cncr.27398

**Published:** 2012-01-17

**Authors:** Bradford S Hoppe, Romaine C Nichols, Randal H Henderson, Christopher G Morris, Christopher R Williams, Joseph Costa, Robert B Marcus, William M Mendenhall, Zuofeng Li, Nancy P Mendenhall

**Affiliations:** 1 University of Florida Proton Therapy InstituteJacksonville, Florida; 2 University of Florida Department of SurgeryJacksonville, Florida

**Keywords:** prostate cancer, proton therapy, radiation therapy, outcomes, toxicity

## Abstract

**BACKGROUND:**

This study sought to evaluate patient-reported health-related quality of life following proton therapy for prostate cancer in men ≤60 years old.

**METHODS:**

Between August 2006 and January 2010, 262 hormone-naive men ≤60 years old were treated with definitive proton therapy for prostate cancer. Before treatment and every 6 months after treatment, patients filled out the Expanded Prostate Index Composite (EPIC) and the International Index of Erectile Function (IIEF) questionnaires. Potency was defined as successful sexual intercourse in the prior month or an EPIC sexual summary (SS) score ≥60.

**RESULTS:**

Median follow-up was 24 months; 90% of men completed follow-up EPIC forms within the last year. For EPIC urinary, bowel, and hormone subscales, the average decline from baseline to 2 years was ≤5 points, except for bowel function (5.2 points). SS scores declined 12.6 points after 2 years. Potency rates declined by 11% from baseline at 2 years, but 94% of men were potent with a baseline IIEF > 21, body mass index < 30, and no history of diabetes. At 2 years after treatment, only 1.8% of men required a pad for urge incontinence. On multivariate analysis, factors associated with a significant decline in SS score were mean penile bulb dose ≥40 cobalt Gy equivalents (*P* = .012) and radiation dose ≥80 cobalt Gy equivalents (*P* = .017); only diabetes was significantly associated with impotence (*P* = .015).

**CONCLUSIONS:**

Young men undergoing proton therapy for treatment of prostate cancer have excellent outcomes with respect to erectile dysfunction, urinary incontinence, and other health-related quality of life parameters during the first 2 years after treatment. Longer follow-up is needed to confirm these findings. Cancer 2012. © 2012 American Cancer Society.

Erectile dysfunction (ED) is a critical health-related quality of life (HRQoL) outcome in men treated for prostate cancer and has been associated with depression and significant distress.^1^, ^2^ Many effective treatment options are available to men with prostate cancer, including surgery, external-beam radiotherapy (EBRT), and brachytherapy, and some men select treatment based on perceived side effect profiles, frequently focusing on urinary incontinence and ED.

Several studies have investigated and compared HRQoL factors following various treatments for prostate cancer.^3^-^6^ These studies have consistently found a more significant decline in ED following surgery than after EBRT. The median age of patients receiving surgery is typically significantly lower than that of patients who receive EBRT; in addition, the EBRT population also includes less healthy men who are not candidates for surgery.^3^, ^6^ Thus, reported ED outcomes following EBRT are generally based on an older, less healthy cohort with lower baseline sexual function and may not reflect outcomes attainable with EBRT in a younger, healthier population of patients with prostate cancer.

Proton therapy (PT) is a highly conformal radiotherapy modality that delivers much less radiation dose to nontargeted normal tissues, such as the bladder and rectum, than conventional radiation therapy. Moreover, comparative dosimetry studies have documented lower doses to nontargeted tissues with PT compared with intensity-modulated radiotherapy,^7^ providing a rationale for the very low rates of genitourinary and rectal toxicity that have been observed in clinical studies^8^ and the expectation of a lower rate of second malignancy in younger men with prostate cancer.^8^-^10^

Although patient-reported ED and urinary incontinence following PT have been studied,^11^, ^12^ none have investigated outcomes using the more commonly used Expanded Prostate Cancer Index Composite (EPIC) questionnaire, which can easily be compared with outcomes after other prostate cancer treatments. Furthermore, no study has evaluated ED in a cohort of young men treated with EBRT, where interactions from medical comorbidities are less likely to influence ED outcomes and for whom baseline sexual function would be similar to “typical” surgical patients.

This study investigates patient-reported HRQoL outcomes (with an emphasis on sexual outcomes) through use of the EPIC questionnaire in a young cohort of patients (≤60 years old) who received definitive treatment with PT alone.

## MATERIALS AND METHODS

### Patients

This study was approved by the University of Florida Institutional Review Board (IRB) and included men aged 60 years or less who were treated with definitive PT alone for prostate cancer. These men were all treated on an IRB-approved outcome tracking protocol and may also have been enrolled on 1 of 3 IRB-approved treatment protocols open between August 2006 and January 2010. Patients were excluded if they received hormone therapy (n = 28) or had less than 6 months of follow-up (n = 7). In total, 262 patients were included in the study.

HRQoL parameters were captured prospectively before the start of definitive treatment, every 6 months for the first year, and then every 6 to 12 months annually following treatment, using the 50-item EPIC questionnaire,^13^ the 5-item International Index of Erectile Function (IIEF), and the International Prostate Symptom Score. If a patient was unable to return for follow-up to fill out the questionnaire, the questionnaire was mailed to the patient or the patient filled out the questionnaire through a secure online medical records portal. For the purposes of this study, if a patient did not have 12-month follow-up, his 18-month HRQoL was used instead for assessment at 1 year. If a patient did not have 24-month follow-up, his 30-month or 36-month HRQoL was used. The EPIC summary and subscales were then calculated and reported using a scale of 0 to 100, with higher scores indicating better outcomes. IIEF scores were calculated and reported by a scale of 5 to 25, with higher scores indicating better outcomes. For the purposes of this study, a strict definition of potency was defined as “having successfully engaged in sexual intercourse at least once in the past month” or an EPIC sexual summary score of ≥60.^14^ Patient baseline characteristics and medical comorbidities that could impact ED were extracted from the patient's initial history at consultation.

### Simulation, Planning, and Treatment

The University of Florida Proton Therapy Institute simulation, planning, and treatment guidelines for prostate cancer have previously been published.^15^ In brief, after having 3 to 4 visicoil fiducial markers placed within the prostate by transrectal ultrasound guidance, patients were simulated on a Philips Brilliance computed tomography (CT) big-bore simulator (Philips Healthcare, Andover, Mass). Thirty minutes before simulation, patients voided, then drank 420 cm^3^ (15 ounces) of water. Patient position was secured with a vacuum-locked body mold. Supine positioning was typically used. Saline (100-200 mL) was instilled into the rectum or a rectal balloon was used to stabilize the prostate position.

Immediately after CT simulation, a magnetic resonance imaging (MRI) scan was obtained on a Philips Panorama 0.23T open-MRI system. The CT and MRI images were fused using the Philips Pinnacle AcQSim3 virtual simulation workstation, and imported into the Varian Eclipse treatment planning system (Varian Medical Systems, Palo Alto, Calif). Prostate and seminal vesicle targets along with the penile bulb were contoured by the treating physicians. Normal tissues, including the bladder, rectum, bowel, and femoral heads, were manually contoured by dosimetrists and confirmed by the treating physician. A planning target volume (PTV) was constructed from the prostate and/or seminal vesicles with margins of 4 mm in the antero-posterior and lateral directions, and 6 mm in the superior-inferior direction. Dosimetric specifications required that 95% of the PTV receive 100% of the prescribed dose and 100% of the PTV receive at least 95% of the prescribed dose. Patients were treated with double-scatter PT with right and left lateral (or slightly oblique) field arrangements with customized brass apertures and compensators. Image-guided treatment was performed by using orthogonal kilovolt imaging for fiducial localization. Depending on protocol, patients were treated either with 2 cobalt Gy equivalents (CGE) per fraction to a total dose of 76-82 CGE or at 2.5 CGE per fraction to a total dose of 70-72.5 CGE.

### Statistics

SAS and JMP software were used for all statistical computation (SAS Institute, Cary, NC). A repeated-measures analysis of variance was used to determine whether there was a statistically significant average increase or decrease in EPIC scores between baseline and 24 months after treatment. A likelihood ratio chi-squared test provided the same repeated measures assessment of potency frequencies between baseline and 24 months after treatment. The nonparametric version of the chi-squared test (Fisher exact test) was used for the analysis of the categorical endpoints of potency and 2 dichotomized versions of delta change from baseline EPIC sexual summary score. The 2 dichotomized EPIC sexual summary scores were accomplished by comparing each patient's last available sexual summary score to values equivalent to 0.5 standard deviation (considered clinically relevant in other HRQoL studies)^16^, ^17^ and 1 standard deviation lower than the baseline sexual summary mean. The last available follow-up was also used to determine potency.

## RESULTS

The median follow-up for the entire cohort was 24 months (range, 6-53 months). Thus far, only one patient has developed a biochemical recurrence; he had high-risk prostate cancer (stage cT3a, Gleason score of 8, and pretreatment prostate-specific antigen level of 33) and refused hormone therapy as part of definitive treatment. A separate patient died from an asthma attack 9 months after treatment. A total of 90% of patients contributed follow-up within 1 year of analysis. The proportion of responses at baseline, 6 months, 1 year, and 2 years for the EPIC questionnaire were available for 97%, 96%, 91%, and 87% of patients, respectively.

Patient-, disease-, and treatment-specific details for the patient cohort are summarized in Table 1. The median age was 56 years (range, 41-60 years) and 87% were caucasian. The median height, weight, and body mass index (BMI) were 180 cm (range, 160-198 cm), 87.7 kg (range, 60.9-171.8 kg), and 27.7 kg/m^2^ (range, 20.2-50.2 kg/m^2^), respectively. At baseline, 41 men (16%) admitted to using phosphodiesterase-5 inhibitor.

**Table 1:** Patient, Disease, and Treatment Characteristics

Characteristic	N	%
**Marital status**		
Married	201	77
Divorced/separated	32	12
Single	21	8
Widowed	2	1
Unknown	6	2
**Mood disorder**		
Depression	20	8
Anxiety	19	7
None	223	85
**Diabetes**		
Yes	17	6
No	245	94
**High cholesterol**		
Yes	106	41
No	156	59
**Hypertension**		
Yes	95	36
No	167	64
**Cardiac disease**		
Yes	22	8
No	240	92
**T classification**		
T1c	205	78
T2a	44	17
T2b	10	4
T2c/T3a	3	1
**Gleason score**		
5	2	1
6	167	64
7	88	33
8	5	2
**Prostate-specific antigen**		
<10	236	90
10 to 20	24	9
>20	2	1
**Risk group**		
Low	156	60
Intermediate	100	38
High	6	2
**Radiation dose**		
70-72.5 CGE at 2.5 CGE/fraction	40	15
76-78 CGE at 2 CGE/fraction	183	70
80-82 CGE at 2 CGE/fraction	39	15

Mean EPIC scores with standard deviations over the first 2 years and at last follow-up are listed by subscale in Table 2. For urinary, bowel, and hormone categories, the average absolute reduction from baseline to 2 years was ≤5 points, except for bowel function (5.2 points). Urinary subscores did not substantially change. Urinary incontinence scores only declined from 95.8 at baseline to 92.2 at 2 years. In response to the specific urinary incontinence questions from the EPIC at 2 years, 1.8% of men used a pad to manage urge incontinence.

**Table 2:** Expanded Prostate Cancer Index Composite (EPIC) Scores Over Time in Our Patient Population (and Standard Deviations in Parentheses)

	Baseline	6 mo	1 y	2 y	Last Follow-Up	*P*
Urinary summary	91.6 (8.5)	88.9 (11.6)	87.3 (12.4)	88.5 (11.6)	88.6 (11.5)	<.001
Urinary function	97 (6.9)	94.5 (9.5)	93.4 (10.3)	94.2 (9.5)	94 (9.9)	.0006
Urinary bother	87.7 (11.9)	84.9 (14.7)	82.9 (15.8)	84.4 (15.0)	84.7 (14.7)	.0002
Urinary incontinence	95.8 (9.2)	93.8 (11)	92.5 (13.2)	92.2 (13.0)	92 (12.9)	.0418
Urinary irritative/obstructive	90.2 (9.6)	87 (13.6)	85.6 (14.7)	87.5 (12.7)	87.8 (12.7)	.0002
Bowel summary	95.1 (5.8)	91.7 (10.4)	89.0 (11.7)	90.3 (10.8)	90.8 (10.3)	<.0001
Bowel function	96 (6.9)	91.9 (12.5)	88.9 (14.6)	90.8 (9.3)	90.9 (12.5)	<.0001
Bowel bother	94.1 (6.4)	91.7 (9.5)	89.3 (10.3)	89.7 (13.7)	91 (9.4)	<.0001
Sexual summary	75.5 (17.8)	67.7 (20)	64.4 (22.0)	62.9 (22.6)	62.7 (22.1)	<.0001
Sexual function	71.9 (17.1)	64.2 (18.3)	61.7 (20.7)	60.6 (20.5)	59.7 (20.5)	<.0001
Sexual bother	84.3 (22.1)	75.2 (26.9)	71.0 (27.8)	69.1 (30.2)	70 (28.4)	<.0001
Hormone summary	92.6 (9.2)	90.7 (10.2)	91.7 (9.7)	91.8 (9.2)	91.1 (9.7)	.7673
Hormone function	89.9 (11.3)	88.2 (12)	89.2 (11.3)	89.8 (10.9)	88.9 (11.5)	.9567
Hormone bother	94.5 (8.8)	92.9 (9.9)	93.8 (9.3)	93.6 (8.6)	93 (9.3)	.8169
Number of potential patients	262	262	261	195	262	–
Number of patients with data	255	243	237	170	262	–
Percent of patients with data	97%	93%	91%	87%	100%	–

**Figure 1:**
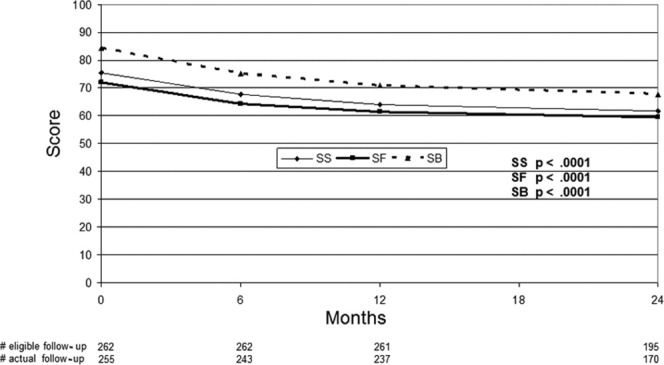
EPIC sexual scores over time are shown. SB indicates sexual bother; SF, sexual function; SS, sexual summary.

The sexual summary, sexual function, and sexual bother scores reported over time are presented in Figure 1. The largest decline in the EPIC sexual summary score occurred within the first year after treatment, with a mean drop of 11.1 points, whereas the mean drop was 12.6 points from baseline to 2 years.

**Figure 2:**
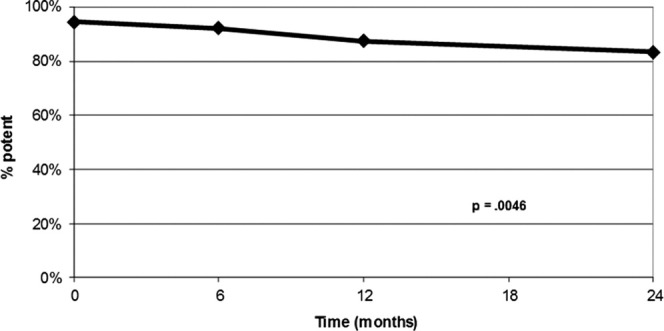
Incidence of potency over time is shown.

The incidence of potency (defined earlier) is depicted for all patients (Fig. 2). Potency rates declined by 11% from baseline over 2 years. In a subgroup of men without diabetes, BMI < 30, and a baseline IIEF > 21, 94% were potent 2 years after treatment.

The degree of ED as defined by the IIEF-5 score (none, >21; mild, 17-21; mild-moderate, 12-16; moderate, 8-11; severe, 5-7) at baseline and at last follow-up is shown in Table 3. The rates of “no ED” or “mild ED” remained high 2 years after treatment (73%) compared with baseline levels (86%). The number of patients actively engaging in sexual intercourse at least weekly only dropped by 11% at 2 years. In addition, answers to selected questions from the EPIC at baseline through last follow-up are also listed in Table 3.

**Table 3:** Distribution of Answers to Specific IIEF and EPIC Questions Over 2 Years

Quality of Life Question	Baseline	6 mo	1 y	2 y
**Erectile dysfunction determined by IIEF score**				
No Problem	69%	57%	48%	45%
Mild ED	17%	21%	25%	28%
Mild to Mod ED	9%	10%	10%	12%
Moderate ED	1%	3%	7%	4%
Severe ED	0%	3%	4%	4%
Not sexually active	5%	6%	4%	8%
**How many pads per day did you use to control leakage during the last month?**				
None	100.0%	99.6%	99.2%	98.2%
One or more pads	0.0%	0.4%	0.8%	1.8%
**During the last month, how often did you have sexual intercourse?**				
Not at all	14%	14%	18%	18%
<Weekly	23%	28%	29%	30%
≥Weekly	63%	58%	53%	52%
**Overall, how big a problem has your sexual function been for you over the last month?**				
No problem	65%	48%	37%	39%
Very small problem	19%	21%	22%	20%
Small problem	7%	14%	17%	15%
Moderate or big problem	10%	17%	24%	26%

ED indicates erectile dysfunction; EPIC, Expanded Prostate Cancer Index Composite; IIEF, International Index of Erectile Function.

The results of univariate and multivariate analyses are listed in Table 4 for sexual summary score and also for potency. On multivariate analysis, no factor was significant for a decline of 50% of baseline standard deviation. Two factors were significant for a decline by baseline standard deviation or greater, including penile bulb mean dose ≥ 40 CGE (*P* = 0.012) and PT dose ≥ 80 CGE (*P* = 0.017). Multivariate analysis for potency was significant for only diabetes (*P* = 0.015).

**Table 4:** Univariate and Multivariate Analysis for Decline in SS Score and Potency

	SS Decline ≥ 50% SD		SS Decline ≥ SD		Potency	
Factor	UVA	MVA	UVA	MVA	UVA	MVA
Age	0.395	–	0.674	–	0.296	–
Body mass index	0.101	0.088	0.269	0.303	0.028	0.211
Marital status	1.000	–	0.743	–	0.465	–
Mood disorder	0.482	–	0.570	–	0.562	–
Dose ≥ 80 CGE	0.482	0.890	0.036	0.017	1.000	0.116
Dose/fraction 2.5 CGE vs 2	0.725	–	0.708	–	0.779	–
Risk (low vs intermediate/high)	0.699	–	0.891	–	0.300	–
Smoker ≥ 10 pack years	0.882	–	0.269	–	1.000	–
Drinks > 7/wk	0.481	–	0.654	–	0.496	–
Bulb mean dose ≥ 40 CGE	0.439	0.233	0.020	0.012	0.801	0.101
Diabetes	0.785	0.674	0.074	0.169	0.015	0.015
High cholesterol	0.522	–	0.056	–	1.000	–
Hypertension	0.794	–	1.000	–	0.202	–
Cardiac disease	0.825	–	0.234	–	1.000	–
Alpha blocker	0.393	–	0.254	–	1.000	–
Pretreatment IIEF < 22	0.310	–	0.877	–	0.001	0.093
Testosterone < 300	1.000	–	0.879	–	1.000	–

CGE indicates cobalt Gy equivalent; IIEF, International Index of Erectile Function; MVA, univariate analysis; SD, standard deviation; SS, sexual summary; UVA, univariate analysis.

## DISCUSSION

Men undergoing EBRT as definitive treatment for prostate cancer have traditionally been older and have had more medical comorbidities (precluding surgery) than men undergoing prostatectomy. Considering the comparatively favorable cure rates emerging from long-term studies of radiotherapy in patients with prostate cancer, younger men are considering radiotherapy as an option due to concerns about urinary incontinence and ED following prostatectomy. The present study investigated HRQoL outcomes with an emphasis on urinary incontinence and erectile function in younger men (≤60 years old) following definitive PT and found only mild changes in the urinary, bowel, hormone, and sexual scores following treatment. Although erectile function appeared to be affected by PT, the EPIC sexual summary scores demonstrated an average decline of only 12.6 points over a 2-year period. Furthermore, potency rates as defined in this study remained high, with 90% of all men and 94% of patients with high pretreatment potency, no obesity, and no diabetes remaining potent 2 years following treatment. Urinary continence levels were also well maintained, including only a 3.6-point decline over 2 years and only 1.8% of men requiring a pad at 2 years (no diapers).

HRQoL studies that evaluated the various prostate cancer treatments through use of the EPIC questionnaire have recently gained in popularity. In the landmark study, Sanda et al^3^ administered the shortened EPIC-26 questionnaire to patients at 0, 2, 6, 12, and 24 months after treatment for prostate cancer with either surgery, EBRT, or brachytherapy. The study demonstrated a substantial decline from baseline sexual summary score and urinary incontinence score in patients at 2 years after undergoing surgery, but only a mild to moderate decline in sexual score and a minimal decline in urinary incontinence score in patients who received either EBRT alone or brachytherapy alone. Brachytherapy and radiotherapy alone did, however, lead to mild increases in urinary irritation or obstructive symptoms and bowel symptoms not seen with surgery. Pardo et al demonstrated similar outcomes after evaluating EPIC scores 3 years after treatment of prostate cancer with surgery, brachytherapy, or EBRT in a cohort of patients from Spain.^6^

Despite the higher radiation doses delivered with PT in our study, the results seen with the EBRT groups in the studies of Sanda et al and Pardo et al are similar with respect to urinary incontinence, urinary irritation/obstructive score, bowel score, and hormonal score. Importantly, Sanda et al reported the use of pads for the management of incontinence at 2 years in 20% of patients following prostatectomy, 5% following EBRT, and 8% following brachytherapy. The outcomes after EBRT are similar to those that were found in our study with a rate of 1.8%. Although the sexual outcomes were better with PT in the present study compared with the other radiation modalities reported in the studies by Sanda et al and Pardo et al, this finding may be due to selection bias. The PT group was made up of younger men with higher baseline sexual function and, thus, we might expect corresponding higher sexual summary scores in follow-up as well. Nevertheless, the data is valuable for prediction of important HRQoL outcomes in younger men facing treatment decisions.

Although the sexual data is hard to compare to other radiotherapy data sets, due to both the high baseline function in this study and young age of the patients, comparisons with surgical data are reasonable. In fact, in the study by Sanda et al, the baseline sexual score was 80, but it dropped to 40 at 2 years following nerve-sparing surgery (and declined to 20 after non–nerve-sparing surgery). Similarly, in the study by Pardo et al, the EPIC sexual summary score of the prostatectomy patients dropped from 67 at baseline to 40 at 2 years after nerve-sparing surgery and 18 after non–nerve-sparing surgery. This contrasts considerably with the results from our study with PT, where baseline function only declined from 75.5 to 62.9 at 2 years. In addition, the number of men engaging in sexual intercourse at least weekly only dropped by 11% (from 63% at baseline to 52% at 2 years) in the 2 years following PT. Thus, preservation of sexual function after PT compares favorably with reported surgical experiences in the first 2 years following treatment.

Despite the excellent outcomes at 2 years, there does appear to be some clinically relevant decline in sexual function and potency following PT, potentially attributable to factors other than increasing age. The multivariate analysis suggests a possible dose-related injury to the penile bulb, because mean dose to the penile bulb ≥40 CGE was associated with a decline by greater than the standard deviation in sexual summary scores. Other studies of penile bulb dose with standard EBRT and ED have yielded mixed results. The study by van der Wielen et al^18^ found no correlation between ED at 2 years and dose to either the crura or the penile bulb in a study of 96 patients treated with doses of 68 to 78 Gy. However, Mangar et al^19^ reported a significant correlation at 2 years between the penile bulb dose-volume histogram (DVH) and ED in 51 men with baseline potency who had received 3 to 6 months of hormonal treatment and 64 or 74 Gy of radiotherapy. Dose received by 90% of the penile bulb (D90) > 50 Gy was significantly associated with ED (*P* = 0.006). Likewise, in a study by Wenicke et al^20^ of 29 men who received 66.6 to 79.2 Gy of 3-dimensional conformal radiotherapy, higher penile bulb doses (D30, D45, D60, and D75) were associated with an increased risk of ED. Finally, in an analysis of the Radiation Therapy Oncology Group 9406 trial,^21^ 158 men who were potent at the start of treatment were evaluated in follow-up, and those who received a penile dose of >52.5Gy had a greater risk of impotence (*P* = .039). The data from these different studies are not entirely consistent but suggestive of dose-related penile bulb injury as a mechanism for radiation-induced ED. More prospective studies with penile bulb dose tracking will be required to determine how reliable this parameter is for predicting preservation of sexual function.

The outcomes reported here are similar to those reported by investigators from the Massachusetts General Hospital, Boston, Massachusetts, in men receiving PT.^12^ Coen et al demonstrated small increases in bowel dysfunction and incontinence with more pronounced changes in sexual dysfunction. Their study, however, used the Prostate Cancer Symptom Indices, which cannot be directly compared with the EPIC data available from contemporary surgery and brachytherapy series and included a much older patient cohort with poorer baseline erectile function.

A major limitation of studies on sexual potency is that ED function is subjective, defined differently in various studies, and clearly affected by non–treatment-related factors. Many studies have included patients who received hormone therapy, which can have a negative effect on ED. Other medical comorbidities, such as diabetes, obesity, and cardiac disease, may not have been assessed, and baseline sexual function may not have been considered. One strength of the present study is in its highly selected cohort of younger patients (≤60 years old) with known baseline function and comorbidities at the time of treatment. In addition, only a small fraction (10%) of patients in the current study have not completed a follow-up in the last 12 months, so the potential impact on outcomes from missing data is minimal. Furthermore, the percentage of patients who responded at 1 year (91%) and 2 years (87%) are similar to the response rate from Sanda et al (92% at 1 year and 87% at 2 years)^3^ and Pardo et al (88% at 1 year and 85% at 2 years).

### Conclusions

Young men undergoing PT for definitive treatment of prostate cancer have excellent outcomes with respect to ED and urinary incontinence. Attempting to keep the mean penile bulb dose to <40 CGE may further improve outcomes. PT may offer young men an excellent treatment option, with a lower risk of ED and urinary incontinence than with surgery. Further follow-up of this cohort of patients is needed to confirm long-term outcomes.
